# Milk traits characterization and association studies with *DGAT1* polymorphisms in Bagnolese sheep

**DOI:** 10.5713/ab.24.0323

**Published:** 2024-10-25

**Authors:** Maria Giulia Pugliano, Gianfranco Cosenza, Emanuele D‘Anza, Andrea Fulgione, Nicoletta Murru, Marika Di Paolo, Sara Albarella, Vincenzo Peretti, Francesca Ciotola

**Affiliations:** 1 Department of Veterinary Medicine and Animal Production, University of Naples Federico II, Naples, Italy; 2 Department of Agriculture, University of Napoli Federico II, Portici, Italy

**Keywords:** Association Analyses, *DGAT1*, Milk Fat, Milk Protein, *Ovis aries*, Single Nucleotide Polymorphisms (SNPs)

## Abstract

**Objective:**

The Bagnolese sheep is an authochtonous dual-purpose breed (milk and meat) reared in the Campania region, whose milk is used to produce Pecorino Bagnolese cheese. Genetic information on this sheep is extremely limited, especially regarding genes affecting productions. The aim of this study was to investigate milk production traits in Bagnolese sheep and the variability of diacylglycerol acyltransferase 1 (*DGAT1*) gene and its effects on milk production.

**Methods:**

Milk quantity was recorded during the morning milking, while Kjeldahl and Gerber methods were used to assess protein and fat percentage (w/v) of collected milk samples. Two polymerase chain reaction-restriction fragment length polymorphism protocols using *BamHI* and *MspI* endonucleases for genotyping of g.5553C>T and g.8539C>T at *DGAT1* locus, respectively, were set up.

**Results:**

Bagnolese sheep milk shows high fat and protein concentrations. Genotyping revealed a high frequency of the g.5553C and g.8539C alleles (0.56 and 0.95, respectively). The association study between the single nucleotide polymorphism (SNP) g.5553C>T and milk traits showed that animals with the CT genotype had a higher percentage of fat produced per milking than those with the CC and TT genotypes (p<0.01). Similar results were found for protein yield percentage, with CT individuals being more productive than CC individuals (p<0.01).

**Conclusion:**

Bagnolese sheep milk parameters found are associated with high yields in the resulting dairy products. CT genotype at the SNP g.5553 of *DGAT1* has shown a positive association with fat and protein milk yield percentage suggesting it could be considered a marker to improve productions of this breed. Finally, the new genotyping techniques used for this study enable a cheap and reliable characterization of two *DGAT1* SNPs in sheep.

## INTRODUCTION

Bagnolese sheep is an Italian autochthonous dual-purpose (dairy and meat) breed [1] whose milk is used to produce Pecorino Bagnolese, a typical cheese also recognized as a traditional agri-food products (PAT) of the Campania Region. To date, the animals listed in the Official Birth Register (National Livestock Association - ASSONAPA) comprise 9,584 adults, including 9,194 females and 390 males, distributed in 119 farms located mainly in Avellino and Salerno provinces and, marginally, in Benevento province.

As is the case for other native breeds, the survival and spread of the Bagnolese sheep is threatened by the homogenization of farming practices and increasing pressure from globalization. Safeguarding native breeds from both a biological and a cultural and economic point of view is a challenge for animal husbandry, also in view of the environmental problems the sector is facing.

A conscious and targeted safeguard requires knowledge of the production and genetic peculiarities of the treated breed. Currently, phenotypic and genetic information concerning the Bagnolese sheep is extremely limited, especially as regards production traits and genes affecting them. Considering the effect of diacylglycerol-acyltransferase 1 (*DGAT1*) on milk production traits such as fat and protein percentage and milk yield [2] and the fact that Bagnolese sheep milk is used for cheese production, characterizing this gene could provide valuable information about the genetic biodiversity of this breed and to improve both the qualitative and quantitative traits of its productions.

Among the enzymes known to influence the lipid metabolism at mammary gland level, the Acyl-CoA: *DGAT1* plays an important role because it catalyzes the final committed step in the formation of triglycerides using diacylglyceroland acyl-CoA as substrates [3].

*DGAT1* is widely expressed in many tissues, with the highest expression levels in the adipose tissue where it controls the triglycerides synthesis, the adipocyte size and the adipose mass. Over-expression of *DGAT1* is, in fact, correlated with the increase in the degree of adiposity [4], such as a down-expression brings to thinness and resistance to diet-induced obesity [5]. Regarding the degree of fat unsaturation, the lack of *DGAT1* expression modifies the fatty acid composition in adipose tissue and skeletal muscle, increasing saturated fatty acids (C16:0 and C18:0) and decreasing monounsaturated fatty acids (C16:1 and C18:1) [6].

In cattle, *DGAT1* became a strong functional candidate for milk fat percentage after [5] described that lactation is absent in knockout mice lacking both copies of *DGAT*1.

Currently, the complete sequences of *DGAT1* gene are available in different livestock species such as Cattle (GenBank accession number AJ318490), Pig (GenBank accession number AY116586), River buffalo (GenBank accession number AY999090), Goat (GenBank accession number LT221856.1) and Sheep (GenBank accession number EU178818.1).

Unlike Cattle and other livestock species few studies have been carried out on *DGAT1* and its role in milk production traits in sheep [7]. In this specie, the *DGAT1* gene is located on chromosome 9 and the coding sequence spans 17 exons. Regarding the gene organization and the length of introns, the *DGAT1* gene structure is similar in all the ruminants [7]. Interesting polymorphisms were described in all dairy species and in particular in Cattle, Buffalo and Goat. Several studies have attempted to associate single nucleotide polymorphisms (SNPs) at this *locus* with different milk production traits (for review see [7]).

In sheep it was observed an association between the SNP EU178818:g.5553C>T located in intron 2 with milk fat content of the Italian breeds Sarda, Altamurana and Gentile di Puglia [2], while association studies showed that a synonymous mutation in exon 17 (EU178818:g.8539C>T, p.487Ala) had a significant effect on some milk traits in Spanish Assaf ewes [8].

In addition to milk qualitative traits, the same polymorphism has been widely investigated to identify associations with meat production traits [7].

In this study steps were taken to: (1) analyse milk production and its variability in Bagnolese sheep, (2) set up two genotyping methods based on polymerase chain reaction (PCR)-restriction fragment length polymorphism (RFLP) to identify the sheep carriers of *DGAT1* g.5553C>T and g.8539C> T transitions, (3) investigate the distribution pattern of the variants for both SNPs of *DGAT1* in Bagnolese sheep, and (4) check their association with milk parameters.

## MATERIALS AND METHODS

### Ethical statement

The Ethical Animal Care and Use Committee of University of Naples Federico II pre-approved all procedures used in this research study (Prot. Nr. PG/2022/0146433). All samples were collected in compliance with the European rules (Council Regulation [EC] No. 1/2005 and Council Regulation [EC] No. 1099/2009). The authors confirm that they have followed EU standards for the protection of animals used for scientific purposes.

### Farms and animals

Eleven farms located in the Avellino, Benevento and Salerno provinces (Campania, Italy) were included in this study.

A total of 252 Bagnolese sheep were used for the present study. Minimum sample size was calculated with the formula from Cochran [9] using the online available “sample size calculator” (https://www.calculator.net/sample-size-calculator.html?type=1&cl=95&ci=10&pp=50&ps=9584&x=Calculate) with CI = 95%; E = 10%; population proportion = 50%; population size 9,584.

The animals were reared following the same traditional management practices of the area: the sheep are left to graze in daylight hours (6/8 h/day) and return to the shed at sunset. The lambs are breastfed for up to 30±5 days postpartum.

Mechanical milking is carried out twice a day, in the morning and in the afternoon, starting from weaning to drying off (at about six months). Information concerning the parity number was also available.

All the animals were enrolled in the Official Birth Register (ASSONAPA) older than 18 months and minimally related.

Blood samples were collected (19 males and 233 females) for genetic characterization. Individual milk samples (100 mL) from 90 sheep from the third lactation, reared in a pilot farm joining the “salvaguardia dell’ambiente e valorizzazione economica della pecora bagnolese” enhancement project, each 15 days for five months (from January to June 2022) were collected in the morning, to evaluate the effect *DGAT1* polymorphisms on the milk parameters.

### Milk analyses

The amount of milk (milk yield) from the morning milking was recorded on the farm. The quality parameters analyzed were protein and fat content.

#### Determination of protein content

The protein content (% w/v) was determined using the Kjeldahl method [10]. Briefly, two grams of milk samples were transferred to a Kjeldahl flask. In the flask, 5.6 g of potassium sulfate powered (Carlo Erba), 0.8 g of copper sulfate powered (Carlo Erba), and 20 mL of sulfuric acid (96 %, Carlo Erba) were added and mixed gently. The mixture was digested in a digestion block until a green solution formed, and then allowed to cool to room temperature. After this, the digestion flask was placed in the distillation equipment and then 50 mL of distilled water and 70 mL of 40% sodium hydroxide solution were added into the digested Kjeldahl flask and distilled for three minutes. Then, the distilled was collected in a Becker and 10 mL of 0.1 N sulfuric acid (H_2_SO_4_) with 100 μL of colorimetric indicator solution (0.1 g of methylene blue and 0.2 g of methyl red dissolved in 100 mL of ethyl alcohol) were added. Finally, the sample was titrated with 0.1 N sodium hydroxide solution (NaOH) from a burette until a faint gray color solution was formed and the burette reading was taken to the nearest 0.01 mL. Blank test was carried out replaced the sample test with distilled water. The percentage of protein in the milk samples were calculated as follows:


$$\text{Protein }(\%)=[(\text{mL of }{\text{H}}_{2}{\text{SO}}_{4}\;0.1\;\text{N}-\text{mL of NaOH }0.1\;\text{N})\times 0.14\times 6.38]/\text{weight of milk sample }(\text{g})$$

#### Determination of fat content

The fat content (% w/v) was measured using the Gerber method [10]. Briefly, an 11 mL milk sample was mixed with 10 mL of Gerber sulfuric acid (90 %, Carlo Erba) in a butyrometer followed by the addition of 1 mL of alcohol isoamyl alcohol (density ranging between 0.808 and 0.818 g/mL). The butyrometer was then sealed with a rubber cork. After sealing, the contents were shaken until the milk sample was completely digested by the acid. The samples were then centrifuged using a Gerber centrifuge (Nova Safety; Funke Gerber, Berlin, Germany) at 65°C for ten minutes. The fat percentage (% w/v) was recorded from the butyrometer reading.

### DNA extraction

DNA was extracted from the blood by use of a Wizard DNA extraction kit (Promega, Madison, WI, USA), following the manufacturer’s instructions.

### Polymerase chain reaction-restriction fragment length polymorphisms for sheep *DGAT1* single nucleotide polymorphisms genotyping

To identify the sheep carriers of *DGAT1* g.5553C>T and g.8539C>T SNPs, two genotyping methods based on PCR-RFLP were developed.

#### Genotyping at the sheep DGAT1 g.5553C>T locus

A 767 base pair (bp) DNA fragment spanning part of the 2th intron and partial exon 3 region of the sheep *DGAT*1 gene was amplified by means of PCR carried out by using iCycler (Bio-Rad, Hercules, CA, USA) with the following primers: DGAT-2F, forward: 5′-TGCATTTCTGAGCCTGTCATC-3′ (nucleotides 5342 to 5362), DGAT-3R, reverse: 5′-AACCGT GCGTTGCTTAAGATC-3′ (complementary to nucleotides 6088 to 6108).

The 25-μL PCR reaction mix included: 100 ng of genomic DNA, 50 mM KCl, 10 mMTris-HCl (pH 9.0), 0.1% Triton X-100, 3 mM MgCl2, 200 nmoL of each primer, dNTPs each at 400 μM, 0.5 U of Taq DNA Polymerase (Promega), and 0.04% bovine serum albumin.

The amplification program consisted of 31 cycles. The first one was characterized by a denaturation at 97°C for 2 min, annealing at 63°C for 45 s and an extension step at 72°C for 2 min. The next 30 cycles involved a denaturation step at 94°C for 45 s, annealing at 63°C for 45 s and extension at 72°C for 2 min with the exception that in the last cycle the extension time was 10 min.

Digestion of 17 μL of each PCR amplification was accomplished with 10 U of *BamH*I endonuclease (Promega) for 5 h at 37°C following the supplier’s directions for buffer conditions.

#### Genotyping at the sheep DGAT1 g.8539C>T locus

For genotyping the SNP g.8539C>T a method based on the *Msp*I endonuclease was devised.

A 365 bp DNA fragment spanning part of the 16th exon to partial exon 17 region of the sheep *DGAT1* gene was amplified with the following primers: DGAT-16F, forward:5′-GCATGATGGCACAGGTGA-3′ (nucleotides 8305 to 8322), DGAT-17R, reverse:5′-GGAGGCAGCTTTCACCAG-3′ (complementary to nucleotides 8652 to 8669).

PCR reaction mix and thermal conditions were performed as reported above. Digestion of 17 μL of each PCR amplification was accomplished with 10 U of *Msp*I endonuclease (Promega) for 5 h at 37°C following the supplier’s directions for buffer conditions.

All primers were designed with DNASIS-Pro version 3.0 software (Hitachi, Tokyo, Japan) using the sheep *DGAT1* sequences as templates (GeneBank Acc. No. EU178818.1). All PCR and digestion products were analyzed directly by electrophoresis in 2% TBE agarose gel (Bio-Rad) in 0.5X TBE buffer and stained with SYBR green nucleic acid stain (Lonza Rockland Inc., Rockland, ME, USA).

### Sequencing analyses

For the validation and confirmation of the PCR-RFLP genotype results, 21 informative samples (5 g.5553C/C, 5 g.5553C/T, 5 g.5553T/T, 5 g.8539C/C, 5 g.8539T/C, 1 g.8539T/T) were amplified, purified with QIAquick columns (Qiagen, Hilden, Germany) and sequenced in outsourcing on both strands by Eurofins Genomics (Ebersberg, Germany) by Sanger technology.

### Statistical analysis

Population genetics and statistical analyses were carried out on the total number of genotyped animals and on the farm animals under research. Allele frequencies and genetic indices of the population analyzed such allele frequencies and genetic indices of the population analyzed such as observed and expected gene heterozygosity and fixation index, were obtained with POPGENE32 software version 1.32 (PopGene: Microsoft Window-Based Freeware for Population Genetic Analysis; University of Alberta, Edmonton, AB, Canada) [11].

Statistical analysis was conducted to estimate the effect of the detected polymorphisms on milk production and milk composition of the analyzed animals considered as a single population.

A mixed repeated-measures model [12] was used with IBM SPSS Statistics software Version 29.0.1.0 to assess the possible relationship between *DGAT1* polymorphisms and the qualitative milk traits under study.

The animals were grouped according to their genotype at the *DGAT1 locus*. Milk production data were considered as repeated measures. The statistical model includes the genotype as a fixed effect (three levels), the fixed effect of parts (two levels, 1st to 2nd and 3rd), the random effect of the animal and the residual error term, as described below:


$${\text{Y}}_{\text{ijk}}=\mu +\gamma_{\text{k}}+\delta_{\text{j}}+{\left(\gamma \delta \right)}_{\text{kj}}+{\text{A}}_{\text{i}}+{ɛ}_{\text{ijk}}$$

where Yijk was the dependent variable indicating response value for animal i (e.g., liters per lactation, average daily milk yield, fat percentage, protein percentage), in lactation phase j, with genotype k; μ was the general mean; γk was the fixed effect of genotype k (three levels); δj was the fixed effect of lactation phase j (two levels); (γδ)kj was the interaction between genotype k and lactation phase j; Ai was the random effect of animal i; ϵijk was the residual error. Values were considered significant at p<0.01. If more than two groups were compared, Bonferroni’s multiple testing was used.

## RESULTS AND DISCUSSION

### Milk analyses

Mean milk yield per lactation (150 days starting after lamb weaning) is 107.00±36.00 kg with an average daily production of 693.20±233.40 mL/die each ewe.

As regard the quality parameters analyzed, mean fat yield % and kg are 9.08±1.26 and 9.82±3.24 respectively, while mean protein yield % and kg are 6.64±0.53 and 7.22±2.11 respectively.

Milk production data recorded in Bagnolese sheep in this study show a high variability which is consistent with the absence of a well-defined selection plan for this trait. However, this breed has a production in line with the European and national average (104.3 and 102 kg/ewe, respectively) [13] and higher than that of other Continents (Supplement 1).

Moreover, Bagnolese sheep can be considered a high milk fat breed, with a mean fat yield percentage (9.08±1.26) that is higher than that of other Italian autochthonous and selected breeds. In contrast, the protein milk content is 6.64±0.53, which is higher than in Sarda sheep and similar to that of other Italian native breeds [2,14,15].

The milk quality parameters observed in Bagnolese sheep support its breeding, which is mainly aimed at Pecorino Bagnolese cheese production. High fat and protein concentrations in the milk are associated with high yields in the resulting dairy products.

### Polymerase chain reaction-restriction fragment length polymorphisms for sheep *DGAT1* single nucleotide polymorphisms genotyping

Two new reliable and cost-effective methods of analysis, based on PCR-RFLP, were set up to identify carriers of the SNPs at position 1,415 of intron 2 (EU178818.1: g.5553C>T) and on the 147th nucleotide of the exon 17 (EU178818.1: g.8539C>T), respectively.

**Figure 1:**
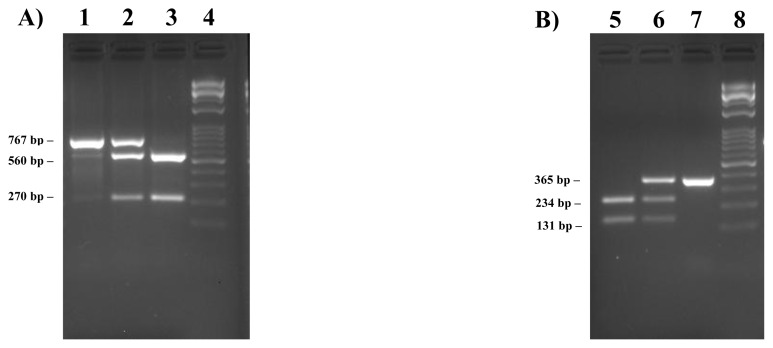
Sheep DGAT1 genotypes detection by PCR-RFLPs. A) Genotyping of the SNP g.5553C>T in intron 2 of Bagnolese sheep *DGAT1* by *BamH*I PCR-RFLP. Lane 1, TT homozygous sample; lane 2, heterozygous sample; lane 3, CC homozygous sample; lane 4, 1kb Opti-DNA Ladder, 0.1 to 10 kb (Applied Biological Materials, Richmond, Canada). B) Genotyping of the SNP g.8539C>T in the DGAT1 exon 17 by *Msp*I PCR-RFLP. Lane 5, CC homozygous sample; lane 6, heterozygous sample; lane 7, TT homozygous sample; lane 8, 1kb Opti-DNA Ladder, 0.1 to 10 kb (ABM). bp, base pair; PCR-RFLP, polymerase chain reaction-restriction fragment length polymorphism; SNP, single nucleotide polymorphism.

The first transition changes a *BamH*I endonuclease restriction site (G/GATC**C**, bold and underlined the polymorphic site) and would allow the identification of T or C-carriers. Therefore, by means of *BamH*I digestion of PCR products, including part of the intron 2 and partial exon 3 (767 bp) of the sheep *DGAT1*, homozygous individuals for g.5553T show one undigested fragment, whereas the same amplicon is restricted into two fragments of 207 and 560 bp in the presence of Citosine at the homozygous status. The restriction pattern of the heterozygous samples shows all 3 restriction fragments (Figure 1; Supplement 2).

Likewise, the transition g.8539C>T removes a *Msp*I endonuclease restriction site (C/**C**GG, bold and underlined the polymorphic site). *Msp*I digestion of a PCR product of 365 bp spanning exon 16 (partial) and 17 (partial), would allow carriers for the presence of Citosine to be identified. As a consequence, the PCR product, uncut in the presence of Timidine, is now restricted to two fragments of 131 and 234 bp. Heterozygous individuals produced a pattern characterized by all 3 restriction fragments (Figure 1; Supplement 3).

While for the first mutation, genotyping protocols are not reported in the literature, for the mutation in the exon 17, various authors apply a genotyping method based on PCR-RFLP using the *Alu*I endonuclease, whose restriction site (AG/C**T**, bold and underlined the polymorphic site) is altered in the presence of Cytosine. As the authors indicate, the *Alu*I PCR-RFLP produces an undigested fragment of 309 bp in the case of C allele and two undigested fragments of 272 and 37 bp for T allele. This last fragment would remain not visible in the gel given the few base pairs (Supplement 4). Therefore, a limitation of the *Alu*I PCR-RFLP method is the suboptimal discrimination of restriction fragments compared to that proposed in this study. In addition to the issues due to electrophoretic pattern resolution, the choice in this research to create a new protocol that involves restriction in the presence of Cytosine was motivated by the very high frequency of this allele observed in most investigated breeds. The use of the *Msp*I endonuclease could provide greater assurance for a more accurate and less ambiguous genotyping, especially where a limitation of the *Alu*I restriction method for the rare allele could be hypothesized due to: low enzyme activity, operator error, incorrect maintenance of incubation temperature, pipetting error, etc.

To validate the results of the two PCR-RFLP protocols, Sanger sequencing was performed and electropherogram analysis confirmed the homo/heterozygosity for each specific marker.

### Genotyping

The Bagnolese sheep population under study was genotyped for the g.5553C>T mutation by *BamH*I -PCR-RFLP. The results of the genotype distribution and allele frequencies for this marker are shown in Table 1.

The results show a higher frequency of the C allele. Based on the expected genotypic frequencies, a statistically significant heterozygote deficiency is observed. The fixation index, which assesses the level of heterozygosity within a population, confirms the excess of homozygotes that could be the sign of an undergoing selection process.

This situation is consistent with the average number of animals per farm (85) and the tendency of farmers to rely mainly on internal breeding, which limits the introduction of breeders from different genetic lines. The g.5553C>T mutation has only been studied to a limited extent. So far, it has been characterized by Scatà et al [2] in three Italian sheep breeds: Altamurana, Gentile di Puglia and Sarda. According to these authors, all three breeds show a higher frequency of the C allele, especially in the Altamurana and Gentile di Puglia breeds (Table 2), which also show a statistically significant association between this SNP and milk fat content.

*Msp*I-PCR-RFLP was used to genotype the g.8539C>T transition at the *DGAT1 locus* of the Bagnolese sheep. The results of the genotype distribution and allele frequencies for this marker are shown in Table 3.

The results show a predominant frequency of the C allele (0.95) being respected Hardy Weinberg Equilibrium.

The presence of cytosine at nucleotide 147 of exon 17 characterizes the remaining ruminant species and is therefore considered the ancestral form of the *DGAT1* gene. However, exceptions to this are ruminants such as the takin (*Budorcas taxicolor*, GeneBank XM_052651466.1) and the sabre-horned oryx (*Oryx dammah*, GeneBank XR_005724623.1), which would be characterized by the presence of T.

Furthermore, the positive association with improved milk processing characteristics [8], such as lactose content, C4:0 fatty acids, C16:1 c9 and the n-6:n-3 ratio, could have favored the maintenance of the high frequency of C allele in the Bagnolese population by unconscious selection of the breeders.

The mutation g.8539C>T compared to the transition g.5553C>T, has been the subject of several investigations in many breeds/genetic types reared in different European and non-European nations and much of the research has been aimed at identifying associations with features of interest such as milk and meat traits. Interestingly allelic and genotypic frequency is similar among most of the analysed breeds (Table 4).

The frequency of the g.8539C allele varies from 1 to 0.95 for the breeds reared in Italy and from 1 to 0.69 for those bred in Spain, Romania, Indonesia, Turkey, Egypt and India (Table 4).

Similarly, Yang et al [16] report that the C allele is always predominant in 4 Chinese breeds (Tan, Ganjia, Oula and Qiaoke), but with a lower C allele frequency (0.62 to 0.78). However, these results contrast with those previously reported by Xu et al [17] for the same (Tan) or other sheep breeds (Small-tailed Han and InnerMongolia) reared in China, where the frequency ratios between C and T alleles were reversed. Similarly, the allele T is reported to be predominant in the Iranian breeds Moghami, Zell and Lori Bakhtiari [18,19]. The exception is the same Iranian breed Lori, characterized by Nanekarani et al [20], where an almost equal frequency of the two alleles is found.

The different allele frequencies observed between different sheep breeds could be caused by several factors like the productive attitude (meat, milk or wool), the different geographical area of origin and breeding or an incorrect genotyping due to misinterpretation of results.

For breeding programs, these findings highlight the importance of considering genetic diversity and selection practices. While the high frequency of the g.8539C allele may be beneficial for current production, it is crucial to maintain genetic diversity to avoid potential inbreeding depression and ensure long-term adaptability.

### Single nucleotide polymorphisms *DGAT1 locus* effect on milk parameters

*DGAT1 locus* in sheep has been widely investigated to identify associations with meat production traits of sheep such as carcass, intramuscular fat content, muscle marbling, fat-tail weight and back fat thickness, meat tenderness [17,23,29] or live weights up to weaning age in lambs [25].

In particular, considering the SNP g.8539C>T association studies with the acidic profile of the carcass showed that meat of heterozygote CT animals have better nutritional characteristics than the CC ones. In fact, Gunawan et al [22] reported a significant association between the CT genotype and a lower content of saturated fatty acids such as stearic acid (C18:0) and peanutic acid (C20:0) compared to the CC genotype in indigenous Indonesian sheep breeds. Moreover, the CT genotype seems associated with a high content of mono-unsaturated fatty acid including oleic acid (C18:1n9c). In the same breeds Amri et al [21] observed that the CT genotype had the highest value of carcass traits compared to CC genotypes. Finally, the CT genotype was associated to significantly heavier birth weight (p = 0.044) compared to CC genotype in Akkaraman male lambs reared in Turkey [25].

On the contrary there is little information about the *DGAT1* gene and its association with milk traits. To our knowledge the only study was carried out by Dervishi et al [8] about the effect of the SNP g.8539C>T in Assaf sheep breed reared in Spain. The association studies showed that lactose, fatty acids C4:0, C16:1 c9, and the ratio n-6:n-3 were affected by this polymorphism. Animals carrying the CC genotype had greater lactose, C4:0 and C16:1 c9 contents and lower ratio of n-6:n-3 compared to the CT ones, but no association was found with the milk fat content and milk yield.

In the 90 lactating Bagnolese sheep here sampled for milk traits association analyses the g.8539C>T exonic SNP was monomorphic for C allele thus the effect of the different genotypes at this *locus* could not be explored in this breed. The CC genotype in literature beyond that associated with positive effect on milk parameters is related to mean carcass weight and dressing percentage [19] and greater fat-tail weight and backfat thickness [18].

The clear predominance of C allele (0.95) in Bagnolese sheep could be a confirmation of its positive effect on milk traits since this breed is mainly used for milk production and only to a lesser extent for meat production.

As regard the intronic SNP g.5553C>T of *DGAT1* gene, the genotyping of the 90 lactating Bagnolese sheep showed that 29 were CC, 36 were CT and 25 were TT. When the association analyses with milk traits was carried out, significant differences were found among the different genotypes (Table 5). In detail, animals carrying the CT genotype produced more fat in percentage per milking if compared to the CC and TT ones (p<0.01). Similar results were found for the percentage of protein yield being CT individuals more productive than CC ones (p<0.01).

These results are in line with those reported by Scatà et al [2] that observed a positive effect of T allele on fat milk yield percentage in Altamurana (n = 37) and Gentile di Puglia (n = 37) sheep.

Currently, it is unclear the reason why this SNP located on intron 2 of *DGAT1* gene has an effect on fat and protein milk yield percentage but it can be hypothesized that it is associated to other causative SNPs in the same or other candidate genes that are still unknown.

The intronic SNP g.5553C>T should be investigated in other breeds, both authoctonous and selected, to confirm the effect observed in this study and determine if the same association with fat and protien milk percentage is present. In particular, selected sheep breeds, which are subjected to genetic improvement, may provide a clearer signal regarding the effect of this SNP.

It should be noted that Bagnolese sheep is not subjected to genetic improvement plans, and as such, historical data on functional controls of milk and meat productions are unavailable. Consequently, the association observed here is based on data from only one lactation.

Additionally, exploring the associations of g.5553C>T and g.8539C>T with other traits, such as meat quality and growth parameters, could provide a more comprehensive understanding of their impacts and guide more informed breeding decisions in Bagnolese sheep conservation plans.

Finally, since all the studied traits are polygenic, future research should investigate other candidate genes to identify favorable haplotypes for improving both the qualitative and quantitative milk traits in Bagnolese sheep.

## CONCLUSION

The main advances in the sheep dairy industry are observed in countries where scientific research supports the sector, such as France and Italy [30]. In this study, the milk production traits of autochthonous Bagnolese sheep have been characterized for the first time, along with the *DGAT1 locus*.

For the first time in sheep, an effect of the SNP g.5553C → T in *DGAT1* gene on milk protein and fat percentage has been detected. These findings underscore the need for continued research into genetic markers and their effects to optimize breeding practices and enhance the overall productivity and sustainability of sheep farming and in particular of autochthonous breeds like Bagnolese sheep. Finally, the development of sensitive, cost-effective genotyping tests for quantitative *loci* in sheep, as demonstrated in this study, will facilitate the optimization of production even in breeds that are not subject to genetic selection.

## Figures and Tables

**Table 1:** Genotype numbers, allele frequencies and population indices observed at the SNP g.5553C>T of *DGAT1 locus* in Bagnolese population (n = 252)

	Genotype numbers			Allele frequency		Population indices		
		
	CC	CT	TT	C	T	Ho	He	FIS
Obs	90	105	57	0.57	0.43	0.42	0.49	0.15
Exp	80.46	124.08	47.46	-	-	-	-	-
χ^2^ = 5.99								
p = 0.01								
d.f. = 1								

SNP, single nucleotide polymorphism; DGAT1, diacylglycerol acyltransferase 1; Ho, observed gene heterozygosity; He, expected gene heterozygosity; FIS, fixation index; Obs, observed; Exp, expected; d.f., degree of freedom.

**Table 2:** Comparison of allele frequency of the SNP at position 1415 of intron 2 of the *DGAT1* gene (g.5553C>T) between the Bagnolese population studied and, the breeds studied by Scatà et al [2]

Breed	No of genotyped animals	Allele frequency		References

		C	T	
Bagnolese	252	0.56	0.44	This research
Altamurana	37	0.75	0.25	[2]
Gentile di Puglia	37	0.68	0.32	
Sarda	34	0.59	0.41	

SNP, single nucleotide polymorphism; *DGAT1*, diacylglycerol acyltransferase 1.

**Table 3:** Number of genotypes, allele frequencies and population indices observed for the SNP at position 147 of exon 17 of the *DGAT1* gene (g.8539C>T) in the studied Bagnolese population (n = 252)

	Genotype numbers			Allele frequency		Population indices		
		
	CC	CT	TT	C	T	Ho	He	FIS
Obs	229	22	1	0.95	0.05	0.09	0.09	0.049
Exp	227.43	11.97	0.63	-	-	-	-	-
χ^2^ = 0.59								
p = 0.44								
d.f. = 1								

SNP, single nucleotide polymorphism; DGAT1, diacylglycerol acyltransferase 1; Ho, observed gene heterozygosity; He, expected gene heterozygosity; FIS, fixation index; Obs, observed; Exp, expected; d.f., degree of freedom.

**Table 4:** Genotype and allele frequencies of the SNP in position 147 of exon 17 of the *DGAT1* gene (g.8539C>T) in different European and non-European breeds

Breed	N. of genotyped animals	Genotype frequency			Allele frequency		References	Country
	
CC	CT	TT	C	T
Bagnolese	252	0.910	0.088	0.001	0.95	0.05	This study	Italy
Altamurana	37	-	-	-	0.94	0.06	[2]	
Gentile di Puglia	37	-	-	-	0.93	0.07		
Sarda	34	-	-	-	1.00	0		
Ansotana	50	0.74	0.18	0.08	0.83	0.17	[9]	Spain
Latxa	36	0.83	0.17	0.00	0.92	0.08		
Romanov	33	0.76	0.18	0.06	0.85	0.15		
Rasa aragonesa	55	0.62	0.33	0.04	0.80	0.20		
Churra	52	0.79	0.19	0.02	0.88	0.12		
Churra tensina	57	0.53	0.33	0.14	0.69	0.31		
Churra lebrijana	50	1.00	0.00	0.00	1.00	0		
Manchega	48	0.73	0.21	0.06	0.83	0.17		
Assaf	402	0.93	0.07	0.00	0.96	0.04		
Compass Agrinac	10	1.00	0.00	0.00	1	0	[21]	Indonesia
Barbados Cross	10	1.00	0.00	0.00	1	0		
Jonggol sheep	15	1.00	0.00	0.00	1	0		
Javanese thin-tailed	15	0.86	0.13	0.00	0.93	0.06		
Compass Agrinac	35	1.00	0.00	0.00	1	0	[22]	
Barbados Cross	36	1.00	0.00	0.00	1	0		
Garut Composite	41	1.00	0.00	0.00	1	0		
Javanese thin-tailed	18	0.90	0.10	0.00	0.95	0.05		
Javanese fat-tailed	20	0.94	0.06	0.00	0.99	0.01		
Barki	25	0.78	0.22	0.00	0.89	0.11	[23]	Egypt
Najdi	25	0.65	0.35	0.00	0.83	0.17		
Harri	25	0.50	0.50	0.00	0.75	0.25		
Lori	118	0.43	0.26	0.31	0.56	0.43	[20]	Iran
Lori-Bakhtiari	152	0.16	0.19	0.65	0.25	0.74	[18]	
Zel	157	0.07	0.24	0.69	0.19	0.81		
Moghani	150	0.04	0.26	0.70	0.17	0.83	[19]	
Turcana	50	0.84	0.12	0.04	0.90	0.10	[24]	Romania
Small-tailed Han	96	0.34	0.09	0.57	0.38	0.62	[17]	China
Tan	94	0.36	0.11	0.53	0.41	0.59		
InnerMongolia	96	0.25	0.02	0.73	0.26	0.74		
Tan	58	0.59	0.38	0.03	0.78	0.22	[16]	
Oula	39	0.51	0.28	0.21	0.65	0.35		
Ganjia	36	0.59	0.22	0.19	0.69	0.31		
Qiaoke	34	0.50	0.23	0.27	0.62	0.38		
Akkaraman	374	0.91	0.09	0.00	0.96	0.04	[25]	Turkey
Imroz	60	0.68	0.27	0.05	0.82	0.18	[26]	
Chios	52	0.52	0.36	0.12	0.70	0.30		
Jaisalmer	42	0.57	0.36	0.07	0.75	0.25	[27]	India
Deccani	38	0.84	0.16	0.00	0.92	0.08		
Muzzafarnagri	50	0.76	0.20	0.04	0.86	0.14		
Mandya	36	0.92	0.08	0.000	0.96	0.04		
Nali	51	0.78	0.20	0.02	0.88	0.12		
Nellore	42	0.74	0.21	0.05	0.85	0.15		
Ganjam	47	0.98	0.02	0.000	0.99	0.01		
Magra	36	0.78	0.19	0.03	0.88	0.12		
Malpura	146	0.86	0.02	0.12	0.92	0.08	[28]	

SNP, single nucleotide polymorphism; *DGAT1*, diacylglycerol acyltransferase 1.

**Table 5:** Effect of genotypes at position 1415 of intron 2 (EU178818.1: g.5553C>T) at *DGAT1* locus on milk yield and composition of 90 Bagnolese sheep

Parameters	Genotypes		

	CC (n = 29)	CT (n = 36)	TT (n = 25)
Milk yield (kg/lactation)	112.51±30.01	109.31±42.35	108.86±22.90
Fat yield (%)	8.43±0.73^B^	9.75±0.98^A^	8.81±1.24^B^
Protein yield (%)	5.78±1.83^B^	6.75±0.36^A^	6.64±0.34^A^
Fat yield (kg)	8.71±3.07	10.45±4.0	9.33±2.45
Protein yield (kg)	6.43±2.14	7.36±2.83	7.07±1.59

A,B p<0.01.

*DGAT1*, diacylglycerol acyltransferase 1.

## SUPPLEMENTARY MATERIAL

**Supplement 1**. Mean milk yield per lactation of ewe in different Continents (FAOSTAT 2022 - https://www.fao.org/faostat/en/#data/QCL) compared with Bagnolese sheep (this study) (data for Oceania are unavailable)

**Supplement 2**. DNA segment comprising the region of the ovine DGAT1 gene between the 2nd intron and exon 3 (partial).

**Supplement 3**. DNA segment comprising the 3′ end of exon 16 (partial), intron 16, exon 17, and 3′ UTR of the ovine DGAT1 gene.

**Supplement 4**. DNA segment between the 16th and 17th exons of the ovine DGAT1 gene (309 bp), amplified for the optimization of the AluI PCR-RFLP method proposed by Xu et al [17].
