# Supplementary material for: Milk traits characterization and association studies with DGAT1 polymorphisms in Bagnolese sheep
Source: Anim Biosci. 2024 Oct 25;38(5):863–72. doi: 10.5713/ab.24.0323 (PMC12062812; doi:10.5713/ab.24.0323)
Supplement: Supplementary file 1 [file ab-24-0323-Supplementary-1.pdf]

Supplement 1: Mean milk yield per lactation of ewe in different Continents (FAOSTAT 2022 - https://www.fao.org/faostat/en/#data/QCL) compared with Bagnolese sheep (this study) (data for Oceania are unavailable)

Country	Milk yield (Kg/ewe)
Europe	104.3
Asia	36.6
Africa	26.5
America	32.7
Bagnolese sheep	107

```
5342  tgcatttctgagcctgtcatctccttgggaagccttccgggtaggggactccatccatgt  5401

5402  cgcctagagaagcatcacttttccacagagccttctgcaaccccgtggggcctgaacct  5461

5462  tgagggtggaggtggtggcccctgcccctgcggagggcagccaggcatctggccccaggc  5521

5522  cactggcaagagctcgttgtgatggaggatcygtcctttgctgctgctgtaggagcggcc  5581

5582  gaggcgggtgggggtgtgagtaggggtggagacccaggcccagcttccccagccctcagg  5641

5642  acaggcccgctctttcccaccaccccaccaagggcgtgggcacaccccgcctctggggat  5701

5702  tgggccccggttggtaagggcggaagcccttgggccggtggcagcgtgcaggcaggcttg  5761

5762  gacttcactggggcttggggctgtcgctgtggccaggggcactgacccgctcagtgggac  5821

5822  ggaggatggctgctgggcagcgggtttcttctgccgtggcggcacaggcacctggggttg  5881

5882  cagttggctccagacgggtgggggctgcgtgcccctgcgcaggcacacaggccataggtg  5941

5942  gggagtctcagagcttggcgtgaggtcccgcagggctgggcctgcaggatggaggctgct  6001

6002  gtcctgagctgtgggtgctggcaggagctggggtgggtgttctggggccgcggctgacag  6061

                                          Exon 3
                                       I  L  S  N  A  R
6062  cattgtgtccctctctctctattgcagATCTTAAGCAACGCACGGTT  6108
```

Supplement 2. DNA segment comprising the region of the ovine *DGAT*1 gene between the 2nd intron and exon 3 (partial). The C>T transition occurring at nucleotide 1415 of intron 2 (EU178818.1:g.5553C>T) is highlighted in red. The *BamH*I endonuclease restriction site (G/GATCC) is underlined and boxed. The forward (DGAT-2F) and reverse (DGAT-3R) primers are shaded in gray.
