# Supplementary material for: Milk traits characterization and association studies with DGAT1 polymorphisms in Bagnolese sheep
Source: Anim Biosci. 2024 Oct 25;38(5):863–72. doi: 10.5713/ab.24.0323 (PMC12062812; doi:10.5713/ab.24.0323)
Supplement: Supplementary file 3 [file ab-24-0323-Supplementary-3.pdf]

Supplement 3

```
           Exon 16
          M  M  A  Q
8305   GCATGATGGCACAGgtgagcagccctggacccccacctgcgagcccaccccgtgggcgca   8364

                                              Exon 17
                                    I  P  L  A  W  I  V  G  R  F  F
8365   gaggctcactcccgtcccatgtccccagATCCCGCTGGCCTGGATAGTCGGCCGCTTCTT   8424

         R  G  N  Y  G  N  A  A  V  W  L  S  L  I  I  G  Q  P  V  A
8425   CCGTGGCAACTATGGCAACGCGGCTGTGTGGCTGTCACTCATCATTGGGCAGCCAGTGGC   8484

         V  L  M  Y  V  H  D  Y  Y  V  L  N  R  E  A  P  T  A487G  T
8485   CGTCCTGATGTACGTCCACGACTACTACGTGCTCAACCGCGAGGCCCCAACAGCYGGCAC   8544

         *
8545   CTGAGCCCCTCCAGGCTGGTTCCCTCAGGGGTGTTGGACTCCTTTGCCTCACCACTTGCT   8604

8605   GCTGTACTGGAGCCTGCCCCCAACCTGGGCGTAGGGGAGGGGCCTGGCTGGTGAAAGCTG   8664

8665   CCTCC   8669
```

Supplement 3. DNA segment comprising the 3' end of exon 16 (partial), intron 16, exon 17, and 3' UTR of the ovine *DGAT*1 gene. The stop codon is symbolized by *. The deduced protein sequence is given above each. The C>T transition occurring at the 147th nucleotide of exon 17 (EU178818.1:g.8539C>T) is highlighted in red. The *Msp*I endonuclease restriction site (C/CGG) is underlined and boxed. The forward (DGAT-16F) and reverse (DGAT-17R) primers are shaded in gray.
