# Supplementary material for: Milk traits characterization and association studies with DGAT1 polymorphisms in Bagnolese sheep
Source: Anim Biosci. 2024 Oct 25;38(5):863–72. doi: 10.5713/ab.24.0323 (PMC12062812; doi:10.5713/ab.24.0323)
Supplement: Supplementary file 4 [file ab-24-0323-Supplementary-4.pdf]

Supplement 4

```
     GCATGTTCCGCCCTCTGG
8278 GCATGTTCCGCC TCTGGGCCTTCACCGGCATGATGGCACAGGTGAGCAGCCCTGGACCCC 8337

8338 CACCTGCGAGCCCACCCCGTGGGCGCAGAGGCTCACTCCCGTCCCATGTCCCCAGATCCC 8397

8398 GCTGGCCTGGATAGTCGGCCGCTTCTTCCGTGGCAACTATGGCAACGCGGCTGTGTGGCT 8457


8458 GTCACTCATCATTGGGCAGCCAGTGGCCGTCCTGATGTACGTCCACGACTACTACGTGCT 8517
       260 bp        AluI restriction site                   49 bp
                              ↓
8518 CAACCGCGAGGCCCCAACAGCYGGCACCTGAGCCCCTCCAGGCTGGTTCCCTCAGGGGTG 8577
                                  ↑
         272 bp                                            37 bp
                        AluI restriction site
                        directed mutagenesis

8578 TTGGACTCC 8586
```

Supplement 4. DNA segment between the 16th and 17th exons of the ovine *DGAT*1 gene (309 bp), amplified for the optimization of the *Alu*I PCR-RFLP method proposed by Xu *et al*. (2008). The C>T transition (Y) occurring at the 147th nucleotide of exon 17 (EU178818.1: g.8539C>T) is highlighted in red. The adopted forward (GCATGTTCCGCCCTCTGG) and reverse primers (GGAGTCCAACACCCCTGA, complement) are shaded in gray. The restriction site of the *Alu*I endonuclease (AG/CT) is underlined, along with the restriction site of *AluI* for site directed mutagenesis. Boxed the sizes of Restriction Fragments taking into account the *Alu*I restriction site. Circled the Restriction Fragments sizes considering the *Alu*I restriction site directed mutagenesis.
